# People living with HIV’s perspectives of acceptability of fee for home delivery of ART: a qualitative study

**DOI:** 10.1186/s12913-023-10533-4

**Published:** 2024-01-17

**Authors:** Xolani Ntinga, Franco Musiello, Thembelihle Pita, Nomagugu Mabaso, Connie Celum, Adam Szpiro, Heidi van Rooyen, Ruanne Barnabas, Alastair van Heerden

**Affiliations:** 1 Centre for Community Based Research, Human Sciences Research Council, Old Bus Depot, Sweetwaters, Pietermaritzburg, KZN 3201 South Africa; 2 Department of Global Health, University of Washington, Seattle, WA USA; 3 Division of Allergy and Infectious Diseases, University of Washington, Seattle, WA USA; 4 Department of Epidemiology, University of Washington, Seattle, WA USA; 5 Department of Medicine, University of Washington, Seattle, WA USA; 6 Department of Biostatistics, School of Public Health, University of Washington, Seattle, WA USA; 7 MRC/Wits Developmental Pathways for Health Research Unit, University of the Witwatersrand, Johannesburg, South Africa; 8 Division of Infectious Diseases, Massachusetts General Hospital, Boston, MA USA; 9 Harvard Medical School, Boston, MA USA

**Keywords:** Antiretroviral therapy, Differentiated service delivery, Fee for ART delivery, People living with HIV, Perspectives, South Africa

## Abstract

**Introduction:**

Significant progress has been made in the HIV response in South Africa; however, gaps remain in ensuring engagement in care to support life-long medication adherence and viral suppression. The National Department of Health (NDoH) has introduced community-based and clinic-based HIV differentiated service delivery (DSD) models to tackle suboptimal adherence and retention in care. Nevertheless, differentiated care models require adaptation to better serve clients who struggle with adherence. There is limited research on the acceptability of fee for home delivery of ART in resource-constrained settings. The current study investigates the acceptability of fee for home delivery of ART among people living with HIV in South Africa.

**Methods:**

Two mixed-gender focus group discussions (FGDs) took place between June and November 2019, consisting of 10 participants in each group. A purposive sampling strategy was employed to identify and select 10 people living with HIV who were ART-eligible but not in care, and 10 people living with HIV who were currently taking ART and in care. Participants were grouped according to their treatment status. A coding framework, informed by a priori categories and derived from topics in the interview guide, was developed and utilized to facilitate analysis.

**Results:**

Participants expressed enthusiasm for having ART home-delivered, as it would save the time spent waiting in long queues at the clinic. However, some participants raised concerns about potential payment difficulties due to high unemployment rates in the community. Some participants believed this would be acceptable, as patients already incur costs for travel and food when visiting the clinic. Participants in both FGDs expressed strong concerns about home delivery of their ART based on fear of accidental disclosure, especially for those who have not disclosed to their immediate families and partners.

**Conclusion:**

Our study suggests that charging a fee for home delivery is an acceptable and innovative approach to supporting PLHIV in maintaining adherence to their medication and remaining in care.

**Supplementary Information:**

The online version contains supplementary material available at 10.1186/s12913-023-10533-4.

## Background

By the end of 2018, in the Eastern and Southern African region, 75–95% of people living with HIV (PLHIV) knew their HIV status, and among those diagnosed, 64% were on antiretroviral therapy (ART), with only 58% achieving sustained viral load suppression [1]. In South Africa, 7.8 million PLHIV, 72% are on ART, and only 66% are virally suppressed [2]. Recent estimates from Statistics South Africa (Stats SA) [3] indicate that PLHIV will reach 8.45 million in 2022. In 2014, the World Health Organization (WHO) started recommending immediate treatment for PLHIV [4–5]. The success of Universal Test and Treat (UTT) in sub-Saharan Africa (SSA) has placed pressure on health services to deliver consistent, high-quality care, including timely access to medication, follow-up of defaulters, and monitoring of drug resistance [6]. Significant progress has been made in the HIV response; however, gaps persist in ensuring consistent engagement in care to support lifelong medication adherence and viral suppression in low-income countries [7]. Although ART is available, suboptimal adherence levels result in poor outcomes, primarily due to a substantial proportion of patients being lost to follow-up (LTFU) [8–10]. Extensive data from SSA since large-scale ART rollout shows suboptimal retention in HIV care [10–12].

In response to the demand for HIV treatment, South Africa’s National Department of Health (NDoH) has implemented community-based and clinic-based HIV differentiated service delivery (DSD) models [13]. DSD models are standard for clinically stable clients that achieve viral suppression and show engagement in care [14]. To address suboptimal retention, it is critical to improve engagement among all PLHIV, especially those who have never been engaged in care and those in a treatment interruption. However, differentiated care models need adaptation to serve clients struggling with adherence. Persons not engaged in care need tailored approaches that are more simplified and client-centered than those who can successfully engage [15].

Thus, DSD models were developed to address the challenge of suboptimal adherence levels leading to poor health outcomes by moving away from a “*one size fits all*” model to focus on tailoring HIV care to individual patient needs [1]. DSD models have predominantly concentrated on de-intensifying HIV care for stable, virally suppressed patients to reduce the burden on the health system and decrease barriers to long-term retention in care for these individuals [16]. Although DSD have been rolled out successfully in several countries since WHO ‘s landmark 2016 guidelines, there is limited research evaluating post-implementation outcomes [16]. In SSA, it is common to find HIV clinics congested with long patient queues, health workers being overburdened by heavy workloads, patients waiting long periods to get assisted, and finding clinics overcrowded because of resource constraints. Out-of-facility innovative DSD approaches, such as fee for home delivery of ART offer an opportunity to overcome clinic-related and transportation costs for PLHIV [17].

It is possible to make healthcare systems better to respond to the needs of people who are not receiving care and to better support those receiving HIV treatments in their quest for better self-management. Creating chances for shared decision-making and mutual respect between PLHIV and the healthcare system for HIV care is essential to this improvement process while also realizing that many reasons for not receiving care are unintentional [18]. However, users’ perspectives on optimal models of HIV care and ART distribution are uncertain [19]. Still, information about patient preferences and views will identify differentiated care models to be prioritized and in which settings. There is limited research on the acceptability and effectiveness of fee for home delivery of ART in resource-constrained settings [17]. In this study, we aim to explore the perspectives on the acceptability of fee for home delivery of ART among PLHIV in South Africa.

## Methods

### Study design

Between June and November 2019, we conducted two (FGDs) to explore the perceptions of clients towards paying a fee for home delivery of HIV care services as contrasted with facility-based care. The FGDs were conducted to gain in-depth insights into views, preferences, and concerns about the proposed service delivery model and facilitate the identification of shared and divergent perspectives among participants.

### Study setting

The study was conducted in the uMgungundlovu district of the KwaZulu-Natal Province of South Africa. The district houses the provincial capital (Pietermaritzburg) and surrounding areas. KwaZulu-Natal has the highest provincial HIV prevalence, with evidence that uMgungundlovu district is among the districts with the highest prevalence in the country at 30% [20]. The district has 57 permanent health facilities serving just over one million residents with 136,481 residents registered in HIV care [21].

### Study population

#### Sampling, and data collection procedure

We purposively recruited 20 participants to participate in the FGDs, from a research database of participants who participated in HIV treatment and prevention and provided consent to be contacted for future studies. This research database is maintained by the Human Sciences Research Council (HSRC). 10 participants were PLHIV who were ART eligible but not in care, and 10 were PLHIV currently taking ART and in care. The participants were adults, 18 years and above, and were grouped according to their treatment status. Two trained bilingual qualitative facilitators LM (Male) and TM (Female) received training on the semi-structured qualitative guide that was developed for this study (Appendix 1) before facilitating the discussions. Both facilitators had prior experience facilitating FGDs and were from the local community. LM and TM facilitated the FGDs using the participants home language (IsiZulu), and a topic guide with open-ended questions. The topic guide explored participants experiences with ART service delivery thus far since their HIV diagnosis. The guide elicited their views, and specific barriers and appeal of clinic and home-based delivery of art medication. It then explored participants’ experiences with community-based ART resupply. It further explored participants’ experiences and perceptions of online purchases with home deliveries to ascertain general delivery feasibility and acceptability. Another topic that was explored is perceptions of fee for pharmaceutical home delivery.

Finally, the discussion explored participants perceptions of the practicalities of offering pharmaceutical home delivery for people living with HIV. All participants provided written informed consents before FGDs which were held in private locations convenient for the participants and were audio recorded. The FGDs lasted between 120 and 150 min.

### Data management and analysis

To maintain confidentiality, participants were provided with codes that they used to identify themselves during the FGD. This also aided the transcription process and referencing quotations. Each FGD had a unique identifying number, which was used to label audio files and transcript documents. Audio recordings were simultaneously transcribed and translated from IsiZulu to English. The transcripts were quality-checked by both the transcriber and a supervising research team member. We analyzed the focus group data using a template analysis approach [22] with templates generated for a priori themes of relevance, looking at the barriers and appeal of clinic vs. home-based delivery of art medication, community-based art resupply, living with HIV perceptions of pharmaceutical home delivery, and practicalities of offering pharmaceutical home delivery for people living with HIV.

Two investigators (XN, and FM) generated an initial coding template within the topical domains relevant to the study. They then engaged in discussions to establish a shared understanding and agreement on this initial framework with the rest of the research team. Any initial differences in coding were identified and resolved through discussion, and the codes were adjusted as needed to create a final coding framework that captured the content of the interviews. Once the template was finalized, XN and FM coded the data set using QSR International’s NVivo 12 qualitative data analysis software. Using coded data, the investigators examined convergences and divergences across interviews to thematically identify the key elements for fee for home delivery of ART, such as convenience of fee for home delivery for people living with ART, willingness to pay for ART, and challenges related to fee for home delivery like unintended disclosure and additional considerations such as linking fee for home delivery for efficient HIV care management.

### Ethical issues

This study was approved by the Human Sciences Research Council Research Ethics Committee (REC 1/21/11/18), in South Africa, and the University of Washington Institutional Review Board, Seattle (STUDY00005739), WA. Permission to undertake the study in the community was sought and granted by the local (uMsunduzi) municipality. Participants individually provided written informed consent after a full explanation of the procedure was provided in their preferred language, and after their questions were answered.

## Results

### Demographics

There were 10 males and 10 females in total across the 2 FGDs. All males had at least a primary level education whereas a total of 3 females had no formal education at all. Table 1 gives the details of the participants in each group.

**Table 1:** Characteristics of the 10 participants making up each FGD

Variable	FGD 1	FGD 2
Age (years)		
Mean (SD)	40.80 (9.647)	33.10 (7.109)
Interquartile Range	(28,57)	(21,46)
Gender		
Male	6 (60%)	4 (40%)
Female	4 (40%)	6 (60%)
Highest level of Education		
None	0 (0.0%)	0 (0.0%)
Primary	0 (0.0%)	3 (30%)
Secondary	10 (100%)	7 (70%)
Tertiary	0 (0.0%)	0 (0.0%)
Employment		
Employed	0 (0.0%)	4 (40%)
Self-employed	0 (0.0%)	4 (40%)
Unemployed	10 (100%)	2 (20%)

### Home delivery of ART

#### Perceived benefits of home delivery

Participants who indicated that they preferred home delivery of their ART, emphasized the convenience home delivery would offer by providing the flexibility of getting medication even when they have other commitments. This was highlighted as an approach that will eliminate the concerns around missed appointments since the medication will be delivered to their home.

*“I think what can help is getting medication delivered at homes because you will get that SMS that by next week they will deliver your medication and you tell them at home that they should expect something if you are not there. It helps because even if you are busy somewhere else, you know that you will be able to find your medication at home, rather than going to the pharmacy and find that they have not yet arrived”* (46-year-old male not in HIV care).

In addition, some participants appeared to be positive about home delivery, as it was perceived to avoid potential HIV stigma associated with clinic visits.

*“I think this strategy of delivering at home will serve us well because some people are just scared to been seen in the clinic queues and having their medication delivered to their homes will make a big difference”* (44-year-old male in HIV care).

### Concerns regarding home delivery

Participants in both FGDs expressed strong concerns about home delivery of their ART particularly due to the fear of accidental disclosure, especially for those who have not disclosed their HIV status to their families.

*“I think a problem with having medication delivered at our homes might be in the cases where for example I did not tell my parents or partner that I am taking certain medication; then during the delivery my privacy or secret might be exposed” (28-year-old female in HIV care).*

Further participants felt it would be almost impossible to arrange ART delivery strategies without people becoming suspicious because seeing people deliver in a healthcare worker uniform would be an indication that someone is sick.

*“I see it as an obvious thing; it is still the same as this thing of nursing assistants. We know that when nursing assistants enters this house, someone is sick. I think it’s too obvious; maybe if there could be a car that is not branded, it would be better because if you come wearing a uniform, we will know that these are nursing assistants and someone is sick here” (P3, 31-year-old male not in HIV care).*

*“I have seen this happening by my house, and now I know that they are taking medication. They come with this document which you need to sign. They have the medicines at hand if they could come up with another way to hide this medication because I know now”* (29-year-old female not in HIV care).

### Fee for home delivery of ART

#### Willingness to pay for home delivery of ART

Participants expressed enthusiasm regarding having their ART delivered and paying a fee for it, indicating the convenience of not having to go to the clinic, saving time spent waiting in long queues.

*“I would like to pay, instead of holding a queue at the clinic, I would rather pay for delivery”* (31-year-old male not in HIV care).

*“I would be willing because it will save me time instead of going to the clinic and spent the whole day there” (46-year-old male not in HIV care).*

### Concerns regarding paying for home delivery of ART

Reservations were raised with the ability to afford the delivery fee.

*“… As people, we do not need to pay money. The same money is the one we need to buy healthy food. We would be happy to have our medication delivered but not pay”* (P6, 27-year-old male not in HIV care).

The major concern participants highlighted against having to pay was the issue of unemployment and they said it might be difficult to raise the funds to pay for the delivery but they still wished to have their ART delivered at home.

*“To be honest, any amount will be a problem for an unemployed person. We will eventually turn into those people who collect waste metals and take them to the scrap yard so that we can be able to get some cash because I have a delivery coming soon. I will start stealing people’s gates, pots at home just to have life”* (P2, 47-year-old male in HIV care).

Despite this, one participant believed that this would not be a limitation as people already incur costs going to the clinic including food costs while waiting at the clinic.

*“I like the idea of paying and also getting medication delivered to us; no one will not be able to pay. We use the money to take transport to go to other clinics because we were running away from clinics nearby. You can take that money and pay for delivery. I believe the price will be reasonable since we get ARVs for free. And it is not like every month I will be paying. P05 mentioned it would be better if they deliver in bulks and not every month”* (37-year-old female not in HIV care).

### Recommendations for facilitating fees for ART home delivery and home delivery processes

#### Linking home delivery to clinic system

Participants expressed that for the home delivery model of ART to work it would need to be linked with the clinic and hospital systems. Participants wanted to benefit from getting their medication delivered to them but also be eligible to go to the clinic or doctor for their annual health check-ups.

*“I think it is a good thing, but only if they are going to link the clinics or hospitals where people will be able to go for their annual check-ups. When your appointment date is close, then they can be able to do the blood check-ups. So, it would be better if they link with those people and not just deliver only”* (39-year-old male not in HIV care).

*“According to how I see and my knowledge, if a doctor says you must come back after six months or after nine months for annual blood check-up that means all these other months the delivery will be happening well, and then you will go to the doctor when the time comes. If the delivery people will be able to contact the facilities and let them know they deliver for these people and also know their appointment dates with doctors”* (46-year-old male not in HIV care).

### Reducing stigma

To potentially mitigate the concern around stigma, participants proposed a possible alternative of making visits infrequent and making use of different vehicles for the deliveries:

### Payment strategies

Participants also came up with possible ways of paying, given the context of unemployment.

*“I would be willing because it will be helping me in the end, but the problem is money”* (P9, 21-year-old female not in HIV care).

*“I think it will be a great idea as a group maybe 4 or 5 of us contribute R10.00 and we pay or let’s say I am not around, I ask my fellow group mates to collect my medication for me, and I pay them later. Or if there are 10 of us, we can contribute R5.00 each which will raise that R50.00. We know that when the delivery comes, we will get our medication because we have raised that amount”* (P8, 51-year-old female in HIV care).

*“I agree with what P8 said earlier, about the idea of creating groups and raising the money needed for the delivery; because raising the money by ourselves will be difficult for some of us. Maybe if there are 20 of us in a group, it will depend on how much Amazon will charge to deliver for us”* (P2, 47-year-old male in HIV care).

## Discussion

Considering the perspectives of PLHIV about the appropriate models of ART delivery in South Africa is critical for improving HIV care and management. The fee for home delivery of ART promises to be an acceptable intervention because of its perceived convenience. However, there are some challenges raised by PLHIV regarding fee for home delivery of ART and possible solutions. These challenges included; ensuring that the home delivery is linked to clinic systems for efficient care management, unintended disclosure with home delivery of ART, and unemployment.

The findings suggest that PLHIV are more enthusiastic about receiving ART deliveries at home than visiting the clinic, given the convenience and flexibility of home delivery. Home delivery was also viewed as enabling one to avoid the HIV stigma associated with clinic visits. Interestingly, studies have shown that home-based ART delivery in comparison to clinic-based ART provision, has similar or even superior health outcomes [23, 24]. In light of this, it is likely that if a barrier to ART adherence is clinic attendance, then this obstacle could be circumvented without compromising health outcomes.

Despite the enthusiasm for the idea of home delivery of ART, some contentious viewpoints were raised regarding willingness to pay for the ART delivery. Some participants indicated they were willing to pay, and others indicated that it would not be possible to pay if one didn’t have the financial means to due to unemployment or if the money could be better used elsewhere. Despite the financial limitation participants were still eager to receive home deliveries of ART. A solution offered by the participants to this financial limitation was to have one delivery at one home for several participants so that the delivery fee could be charged at a single rate which is split evenly across the recipients. A limitation to this could be the possible selling of ARTs by the patient residing at the address before the ARTs are collected by the intended recipient. This may occur as ART can be used to make a recreational drug known in South Africa as “whoonga” or “nyaope” [25].

Despite the concerns related to costs, all participants appeared to eventually concur that given that it costs money to go to the clinic due to travel and food expenses, or perhaps a fee paid to someone who waits in line at the clinic on the patients behalf “holding a queue at the clinic*”*, it would be worthwhile and preferable to pay for delivery, if the amount would be a similar expense as a clinic visit. A solution that was offered for this by the participants was a group delivery. In a paper by Barnabas and colleagues, (2022), in which a randomized trial compared free clinic-based ART provision with a fee for home delivery of ART in South Africa, it was found that 98% of the 80 participants who paid the user fee did achieve viral suppression. There was high acceptability and willingness of fee for home delivery of ART [17]. This concurs with the participants view of paying a fee for ART delivery in the focus groups held in our study. Therefore, it is proposed that if a fee for ART home delivery is charged, it would be acceptable and arguably preferable (in comparison to a clinic visit), so long as the delivery fee is similar to the transport and food and perhaps even other expenses of another clinic visit.

Another challenge raised by participants dealt with stigma and in particular the unintended accidental disclosure of HIV status due to home delivery of ART. This was deemed to be a concern, particularly for those who had not disclosed their HIV status to their cohabitants. Participants indicated that some factors that may result in unintended disclosure included branded vehicles, people delivering the ART whilst wearing healthcare worker uniform, and the same delivery vehicle and person being seen at one’s home repeatedly. These challenges appear to be surmountable with minimal effort at face value by ensuring an unbranded vehicle is used (or perhaps a vehicle that makes delivery of non-medical items e.g., AMAZON), ensuring that medication is delivered by a person not wearing healthcare worker uniform. Using a different delivery vehicle and person every time may be more challenging. However, given that the parcel could be wrapped as suggested by one of the participants it is possible that the delivery could be anything. Nevertheless, similar patient concerns around confidentiality were illustrated in a paper by Hoke and colleagues in 2021, which looked at home delivery of antiretroviral drugs ensured uninterrupted HIV treatment during COVID-19 in Indonesia, Laos, Nepal, and Nigeria [26]. Indeed, the risk of stigma or violence due to inadvertent disclosure of HIV status was presented as a barrier to home delivery acceptance. Despite discreet packaging being used with the deliveries, patients still report subjective fears around confidentiality due to stigma and violence.

There were other challenges raised by the participants which were also accompanied by a possible solution. For example, participants appear to be conscientious about continued access to health care despite receiving ART home deliveries. This was evident in some participants indicating the need for the delivery system to be linked to the health care facilities so that necessary laboratory tests and health care provider check-ups are still done. This challenge appears to go beyond the participants perception and appears to be a challenge in practicality as in a systematic review conducted by Okere and colleagues, a lack of robust monitoring systems was reported as a challenge to DSD interventions [27]. Perhaps this concern could be addressed by timing one of the ART dispensations with a healthcare provider visit to ensure that patients do not merely continue to receive ART deliveries without receiving the necessary health check-ups, however further research would be required.

Our study had one limitation, the overrepresentation of people who were unemployed in our sample, we recognize that exploring client’s perspectives with different sociodemographic characteristics would be an added value.

## Conclusion

Our study suggests that fee for home delivery is acceptable as an innovative approach to supporting PLHIV to maintain adherence to their medication and remaining in care. The enthusiasm for fee-for-service home delivery of ART expressed by participants in this study highlights the potential for this differentiated service delivery model to improve ART adherence by offering convenience and flexibility offered by this method of DSD. Despite the challenges identified, such as concerns around unintended disclosure of HIV status, linking home delivery to clinic systems for efficient care management, and the affordability of delivery fees, particularly for unemployed individuals, our findings suggest that these challenges are not insurmountable. By implementing the proposed solutions, such as discreet delivery methods, group deliveries, and coordinated care with healthcare providers, it is possible to address these concerns and optimize the acceptability and effectiveness of home delivery of ART.

### Electronic supplementary material

Below is the link to the electronic supplementary material.

Supplementary Material 1

## Data Availability

The data sets generated and analyzed during this study are available from the corresponding author upon reasonable request.
