# Supplementary material for: People living with HIV’s perspectives of acceptability of fee for home delivery of ART: a qualitative study
Source: BMC Health Serv Res. 2024 Jan 17;24:88. doi: 10.1186/s12913-023-10533-4 (PMC10792944; doi:10.1186/s12913-023-10533-4)
Supplement: Supplementary file 1 — Supplementary Material 1 [file 12913_2023_10533_MOESM1_ESM.docx]

**The Deliver Health Study**

**PARTICIPANT DEMOGRAPHIC INFORMATION**

1. Participant Date of Birth : _____________________________
2. Participant Gender : Male / Female / Transgendered
3. Focus Group Number : _____________________________
4. Employment : _____________________________
5. Level of Education :______________________________

***______________________________________________________________________________***

***Focus Group Discussion Guide:***

***Delivered Health.***

**INTERVIEW TOPIC 1: BARRIERS AND APPEAL OF CLINIC VS. HOME BASED DELIVERY OF ART MEDICATION?**

- **What are your thoughts about the barriers and appeal of clinic and home based delivery of art medication?**

Probes:

- - Barriers to HIV care and ART resupply at clinic vs. home?
  - Appeal of HIV care and ART resupply at clinic vs. home?
  - Stigma and confidentiality concerns of HIV care and ART resupply at clinic vs. home?

**INTERVIEW TOPIC 2: COMMUNITY BASE ART RESUPPLY**

- **Can you tell me about your experience with community based resupply?**

Probes:

- - If you have any personal experiences, please could you share them with us?
  - Do you know of any other people that have received ART in community settings and what was their experience like?
  - Preference for mobile clinic vs home? Why?
  - Pick-up from CCMDD points?

**INTERVIEW TOPIC 3: HOME DELIVERY**

- **Can you tell me about a time that you ordered something and had it delivered to your house?**

Probes:

- - If not, why not?
  - How did you pay?
  - What would be the best way for you to pay?
    - Cash?
    - Credit Card?
    - Airtime?
    - EFT?
    - Other?
  - What sort of things would you be interested in buying and having delivered to you?

**INTERVIEW TOPIC 4: AMAZON FOR ARTs IDEA**

- **What do you think about the idea of a service provider who will deliver ART medication to you at a time and place that is convenient?**

Probes:

- - Would you be willing to pay for such a service?
  - How much or why not?
  - Where would you like them to deliver and why?
  - What would be the best way to keep in contact with you about delivery details?
    - SMS? App? Phone call?
    - What information would you like to know about your delivery?
  - If you were starting such a business, what would you call it?
    - Where would you advertise?
    - How would you get clients?
    - How would you market your service?
  - What about such a service for medicines other than ARTs?

**INTERVIEW TOPIC 5: AMAZON FOR ARTs FACTORS**

- **Imagine Amazon started offering this service in your community. What do you think they would need to know in order to get people to use the service?**

Probes:

- - What would motivate people to use the service?
  - How could they make it extremely convenient for people?
  - What would they need to do to build trust?
  - How important would privacy be and what could be done to ensure people’s privacy?
  - Would people find such as system complex to understand? What could be done to reduce complexity?
  - Would offering a no cost trial reduce any suspicion that may exist?

**INTERVIEW TOPIC 6: TRANSFERING VALUE**

- **How do people most commonly pay for goods and services in this community?**

Probes:

- - Do people generally have bank accounts?
  - What form of payment to people trust the most?
  - How do people transfer money if they don’t have a bank account?
  - Have you ever heard of Zapper, Snapscan, mPesa or Samsung Pay?
  - Have you ever heard of people using cryptocurrencies such as bitcoin to pay for things?

**INTERVIEW TOPIC 7: HEALTH SYSTEM PERSPECTIVES (Key Informants only)**

- **What do you believe to be the best ART delivery strategy and why?**

Probes:

- - Clinic dispensing?
  - Increase number of month of supply?
  - Community Approaches?
  - Medipost?
  - Home?
  - Community Mobile Clinic?
- **What are people at your levels thoughts and beliefs about the benefits and challenges of home ART delivery?**

Probes:

- - Cost?
  - Patients need to take responsibility for their care?
  - Staffing resources?
  - Other resource shortages?
- **What are the thoughts and beliefs about the benefits and challenges of home ART delivery from people more senior than you?**

Probes:

- - Cost?
  - Patients need to take responsibility for their care?
  - Staffing resources?
  - Other resource shortages?

*Thank the participant and close the interview.*
